# Vaccination of ferrets with a recombinant G glycoprotein subunit vaccine provides protection against Nipah virus disease for over 12 months

**DOI:** 10.1186/1743-422X-10-237

**Published:** 2013-07-16

**Authors:** Jackie A Pallister, Reuben Klein, Rachel Arkinstall, Jessica Haining, Fenella Long, John R White, Jean Payne, Yan-Ru Feng, Lin-Fa Wang, Christopher C Broder, Deborah Middleton

**Affiliations:** 1 CSIRO Livestock Industries, Australian Animal Health Laboratory, 5 Portarlington Road, Geelong, VIC 3220, Australia; 2 CSIRO Biosecurity Flagship, Australian Animal Health Laboratories, 5 Portarlington Road, Geelong, VIC 3220, Australia; 3 Department of Microbiology and Immunology, Uniformed Services University, Bethesda, MD 20814, USA; 4 Program in Emerging Infectious Diseases, Duke-NUS Graduate Medical School, Singapore 169857, Singapore

**Keywords:** Nipah virus, Hendra virus, Henipavirus, Paramyxovirus, Ferret, Immunity, Vaccination, Glycoprotein, Subunit vaccine, Longevity

## Abstract

**Background:**

Nipah virus (NiV) is a zoonotic virus belonging to the henipavirus genus in the family *Paramyxoviridae*. Since NiV was first identified in 1999, outbreaks have continued to occur in humans in Bangladesh and India on an almost annual basis with case fatality rates reported between 40% and 100%.

**Methods:**

Ferrets were vaccinated with 4, 20 or 100 μg HeVsG formulated with the human use approved adjuvant, CpG, in a prime-boost regime. One half of the ferrets were exposed to NiV at 20 days post boost vaccination and the other at 434 days post vaccination. The presence of virus or viral genome was assessed in ferret fluids and tissues using real-time PCR, virus isolation, histopathology, and immunohistochemistry; serology was also carried out. Non-immunised ferrets were also exposed to virus to confirm the pathogenicity of the inoculum.

**Results:**

Ferrets exposed to Nipah virus 20 days post vaccination remained clinically healthy. Virus or viral genome was not detected in any tissues or fluids of the vaccinated ferrets; lesions and antigen were not identified on immunohistological examination of tissues; and there was no increase in antibody titre during the observation period, consistent with failure of virus replication. Of the ferrets challenged 434 days post vaccination, all five remained well throughout the study period; viral genome – but not virus - was recovered from nasal secretions of one ferret given 20 μg HeVsG and bronchial lymph nodes of the other. There was no increase in antibody titre during the observation period, consistent with lack of stimulation of a humoral memory response.

**Conclusions:**

We have previously shown that ferrets vaccinated with 4, 20 or 100 μg HeVsG formulated with CpG adjuvant, which is currently in several human clinical trials, were protected from HeV disease. Here we show, under similar conditions of use, that the vaccine also provides protection against NiV-induced disease. Such protection persists for at least 12 months post-vaccination, with data supporting only localised and self-limiting virus replication in 2 of 5 animals. These results augur well for acceptability of the vaccine to industry.

## Background

Nipah virus (NiV) is a zoonotic virus belonging to the henipavirus genus in the family *Paramyxoviridae*. Hendra virus (HeV) is the only other recognised member of the henipavirus genus [1], although there is increasing evidence of a range of henipa-like and other paramyxoviruses [2-6], particularly in bats which are the reservoir host for both NiV and HeV. HeV and NiV are classified as BioSafety Level 4 (BSL-4) agents, a designation that reflects their ability to cause significant morbidity and mortality in humans as well as the absence of vaccines and post-exposure treatments. Since NiV was first identified [7] outbreaks have occurred in humans on an almost annual basis with case fatality rates generally reported between 40% and 100% [8]. Human-to-human transmission, although not yet observed for HeV [9,10], has been recorded in NiV outbreaks in Bangladesh. Here the local custom of family nursing infected members has resulted in extended exposure to infectious bodily secretions, particularly respiratory fluids [11]. Documentation of human-to-human transmission in this highly virulent group of viruses confirms the possibility of sustained endemic human-to-human transmission and the attendant consequences.

Vaccination is an important component of outbreak preparedness and considerable data has now been accumulated on a subunit vaccine incorporating the G glycoprotein of HeV (HeVsG). The HeVsG vaccine has so far proved effective in preventing disease following exposure to both HeV and NiV. This is as predicted because the G glycoproteins of NiV and HeV share 83% amino acid identity [12] and also host cell receptors - ephrin B2 and ephrin B3 [13-15]. Cats vaccinated with HeVsG produced high levels of neutralising antibody to both HeV and NiV [16]; neutralising antibody to surface glycoprotein has been shown to be particularly effective in protecting against infection with viruses like the henipaviruses that have a viraemic phase [17-19]. Most vaccinated animals not only remained clinically well following exposure to virus but had no evidence of infection, including no boost in vaccinal antibody titres. Vaccination with HeVsG has also been shown to prevent NiV infection in cats and nonhuman primates, and HeV infection in ferrets [16,20-22] and horses. In this last case, HeVsG antigen forms the basis of a commercially available vaccine for HeV - the first vaccine licensed and deployed for use against a BSL-4 agent (Middleton et al., manuscript in preparation). Here we show that ferrets vaccinated with HeVsG were equally protected against NiV disease at 20 days post vaccination as against HeV [22]. Furthermore, protection from disease persisted for over 12 months.

## Results

### Nipah virus challenge of immunized ferrets

The aim of this study was to determine whether a HeVsG based vaccine protected ferrets against disease caused by NiV and the duration of any vaccine-induced protection. Eight ferrets were exposed to an otherwise lethal dose of NiV at either 20 days post vaccination (study 1) or 14 months post vaccination (study 2).

Study 1: Two control ferrets 1–0 and 2–0 were given only adjuvant; 1–0 was febrile on day 5 pi and was euthanized on day 6 pi, while 2–0 was febrile on days 7 and 8 pi and was euthanized on day 10 pi. Two vaccinated ferrets, 4–4 and 7–100 were euthanized prior to exposure to virus for reasons unassociated with the scientific study. The remaining vaccinated ferrets, 3–4, 5–20, 6–20 and 8–100 remained clinically healthy and were electively euthanized on day 41 of the study.

Study 2: Two control ferrets, 9–0 and 10–0 became febrile and exhibited reduced playfulness by day 8 pi; both were euthanized. Ferret 15–100 was euthanized prior to exposure to virus for reasons unassociated with the scientific study. The remaining vaccinated ferrets, 11–4, 12–4, 13–20, 14–20 and 16–100 remained clinically healthy and were electively euthanized on day 455 (21 days pi) of the study.

### Gross pathology, histopathology and immunohistochemistry

Study 1. Control ferrets 1–0 and 2–0 both had histological lesions including widespread deposition of NiV antigen consistent with acute NiV infection as previously described [23]. No significant histological lesions were detected in the remaining 4 ferrets, 3–4, 5–20, 6–20 and 8–100 and NiV antigen was not detected in any of their tissues.

Study 2. Control ferrets 9–0 and 10–0 both had histological lesions including widespread deposition of NiV antigen consistent with acute NiV infection as previously described [23]. No significant histological lesions were detected in the tissues of vaccinated ferrets, 11–4, 12–4, 13–20, 14–20, and 16–100 and NiV antigen was not detected in any of their tissues.

### Viral RNA detection and virus isolation

Viral RNA detection results for study 1 are shown in Table 1. Viral RNA was found in all fluids, swabs and tissues of control ferrets 1–0 and 2–0 and virus was re-isolated from tissues collected from them post mortem. C_t_ results for positive samples and tissues are shown in Table 2. Where virus was isolated from viral RNA positive samples the titre was generally not greater than 10^5^ TCID_50_/ml. These data confirm that virus exposure was sufficient to induce serious infection in the naïve animal. In contrast viral RNA was not detected in any swabs, fluids or tissues collected from the 4 vaccinated ferrets either during the immediate post-exposure period or at post mortem examination; this included ferret 3–4 which had received the lowest dose (4 μg) of HeVsG.

**Table 1:** **Study 1**: **Genome detection in ferrets challenged at day 20 post vaccination**

**Ferret# - dose HeVsG**	**Ab titre to NiV at challenge**	**Eut dpc**	**Genome detected by PCR (dpc)**						**Virus positive tissues**
			**Oral swab**	**Rectal swab**	**Blood**	**Nasal wash**	**Urine**	**Tissues**	
**1-0**	<2	6	PM	PM	PM	PM	PM	13/13	8/13
**2-0**	<2	10	6, 8, PM	8, PM	8, PM	8, PM	PM	13/13	6/13
**3-4**	64, 64	21	-	-	-	-	-	0/13	ND
**4-4**	NC	na	NC	NC	NC	NC	NC	NC	ND
**5-20**	256, 256	21	-	-	-	-	-	0/13	ND
**6-20**	256, 512	21	-	-	-	-	-	0/13	ND
**7-100**	NC	na	NC	NC	NC	NC	NC	NC	ND
**8-100**	256, 512	21	-	-	-	**-**	-	0/13	ND

*Ab* antibody, *Eut dpc* day post challenge that ferrets were euthanized *PM* post mortem, *NC* no challenge, *ND* not done; -, no viral RNA detected, *na* not applicable.

**Table 2:** **C**_**t **_**values in genome positive samples in studies 1 and 2**

**Sample**	**Sample day**	**Genome detected by PCR (C**_**t**_**)**					
		**1**–**0**	**2–0**	**9–0**	**10–0**	**13–20**	**14–20**
**Oral swab**	D6	-	31.4	**-**	32.6	**-**	**-**
	D8	**-**	34.8	**-**	**-**	**-**	**-**
	PM	33.6	30.5	38.0	31.9	**-**	**-**
**Rectal swab**	D6	-	-	-	35.8	**-**	**-**
	D8	**-**	37.5	**-**	**-**	**-**	**-**
	PM	33.3	31.2	**-**	35.4	**-**	**-**
**Blood**	D6	**-**	**-**	**-**	36.6	**-**	**-**
	D8	**-**	32.9	38.3	**-**	**-**	**-**
	PM	30.2	30.1	**-**	26.5	**-**	**-**
**Nasal wash**	D6	**-**	**-**	**-**	29.4	**-**	34.1
	D8	**-**	36.4	**-**	**-**	**-**	32.3
	PM	28.0	29.6	31.9	31.9	**-**	**-**
**Urine**	PM	31.0	29.1	34.7	31.9	**-**	**-**
**Tissues**	PM	^1^18-34.7	18.0-30.9	18.6-36.6	16.4-37.8	^**2**^33.8	**-**

1-0, 2–0 = control ferrets, study 1; 9–0, 10–0 = control ferrets, study 2; 13–20, 13–20 = ferrets receiving 20 μg HeVsG in study 2. *PM* post mortem; -, no viral RNA detected. ^1^Range of C_t_ values in tissues; ^2^bronchial lymph nodes.

Viral RNA detection results for study 2 are shown in Table 3; the pattern of genome detection and virus re-isolation from the control ferrets 9–0 and 10–0 confirmed an otherwise lethal exposure to NiV. Viral genome was not detected in any swabs, fluids or tissues from 3 of the 5 vaccinated ferrets. In one animal (ferret 14–20), viral genome was detected in nasal washes at day 6 and day 8 pi and, in another (ferret 13–20), from the bronchial lymph nodes at post mortem. In neither case was virus re-isolated from these samples. C_t_ results for positive samples and tissues are shown in Table 2. Where virus was isolated from viral RNA positive samples the titre was generally not greater than 10^5^ TCID_50_/ml

**Table 3:** Study 2: Genome detection in ferrets challenged at 434 days post vaccination

**Ferret # - dose HeVsG**	**Ab titre to NiV at challenge**	**Eut dpc**	**Genome detected by PCR (dpc)**						**Virus positive tissues**
			**Oral swab**	**Rectal swab**	**Blood**	**Nasal wash**	**Urine**	**Tissues**	
**9-0**	<4, <4	8	PM	-	PM	PM	PM	13/13	10/13
**10-0**	<4, <4	8	6, PM	6,PM	6, PM	6, PM	PM	13/13	10/13
**11-4**	32, 128	20	-	-	-	-	-	-	ND
**12-4**	32, 64	20	-	-	-	-	-	-	ND
**13-20**	16, 16	20	-	-	-	-	-	1/13	0/13
**14-20**	64, 128	20	-	-	-	6,8	-	^-^	-
**15-100**	NC	na	NC	NC	NC	NC	NC	NC	NC
**16-100**	64, 64	20	-	-	-	-	-	-	ND

*Ab* antibody, *Eut dpc* day post challenge that ferrets were euthanized, *PM* post mortem, *ND* not done, *NC* no challenge; -, no viral RNA detected, *na* not applicable.

### Post-challenge serology

In both studies sera were collected at the time of exposure to virus; day 6, 8 and 10 pi, and at euthanasia. There was no clear cut evidence of an anamnestic antibody response in either group.

## Conclusions

As the human population expands into previously untouched environments there are increasing reports of viruses that have co-existed for long periods of time in their reservoir hosts ‘spilling over’ to emerge as novel human infections. The recent example of such an agent, human immunodeficiency virus (HIV), demonstrates the devastating economic and social impact that these events may have on human populations. Although the virus was not identified until 1981, HIV is thought to have made the initial jump from chimpanzees into humans at the beginning of the 20^th^ century. By 2010, 34 million people were living with acquired immunodeficiency syndrome (AIDS) and in the same year 2.4-2.9 million people were newly infected with HIV. HeV and NiV were also discovered relatively recently – HeV was isolated and identified in 1994 when it caused the death of 20 horses and 1 human [24] and NiV in 1998–9 after an outbreak in pigs and humans in Peninsular Malaysia and Singapore [7]. They were identified as paramyxoviruses but were sufficiently distinct to be assigned to a new genus, *Henipavirus*, within the *Paramyxoviridae*[25,26]. Their large genome was atypical as was their pathogenicity for a range of species, with reported mortality rates in humans of up to 100% for NiV and 57% for HeV. All human cases of HeV infection to date have resulted from close contact with secretions of infected horses either late in incubation period, during terminal illness, or at post mortem examination. There is no known instance of transmission directly to humans from the reservoir host, the bat, or of human-to-human transmission of HeV. In contrast, NiV in Bangladesh can be transmitted directly from bats to people and this has been linked epidemiologically to the consumption of contaminated palm sap; human-to-human transmission also occurs here and is thought to be facilitated by families nursing sick relatives with attendant copious exposure to infected bodily fluids [27]. Repeated spillover events into human (and other animal populations) have been documented for both HeV and NiV [8] suggesting persistence of the environmental circumstances that facilitated the initial emergence event. Spillover is thus likely to be ongoing, and it is conceivable that a future incident with increasing host adaptation might result in establishment of HeV or NiV in human populations, with an impact exacerbated by high mortality rates and no pre-existing immunity.

The sporadic occurrence of viral spillover events creates major challenges for emergency disease preparedness activities. However, in the 15–20 years since HeV and NiV were first identified substantial progress has been made in the development of vaccines and therapeutics for the prevention and treatment of infection with these viruses. The HeVsG vaccine, which prevents HeV disease in horses, is the first to be registered for use against a BSL-4 agent, and a therapeutic monoclonal antibody is currently being assessed for human use [23,28]. While the focus in Australia is on management of the infection risks posed by HeV infection, studies have shown that the HeVsG vaccine provides equally powerful cross-protection against NiV infection in cats and nonhuman primates.

Our current studies indicate that at the time of onset of protection the HeVsG vaccine can reliably protect ferrets from acute NiV disease and also prevent infection – providing so-called sterilizing immunity - consistent with earlier studies using HeV [22]. Here we have also shown, in the first duration of protection study in an animal model, protection from disease persists at least 14 months after vaccination in ferrets. Recovery of viral genome (but not live virus) from the nasal washes of one animal and from the bronchial lymph nodes of another, in the absence of a rise in antibody titre, are consistent with self-limiting local virus replication at a level insufficient to generate an anamnestic immune response or to sustain a transmission event.

The animal numbers reported here are necessarily small due partly to the limitations of the BSL4 facility but also because 3 ferrets were euthanized before challenge leaving 3 groups in the 2 studies with a group size of 1. While no conclusion can be drawn from a group size of 1 there were 3 remaining groups where n = 2, the 20 μg group in both studies and the 4 μg group in study 2. Data from these groups indicated that the vaccine induced protection sufficient to suppress the course of a human pandemic persists for at least 12 months. The data from the reduced groups also support this conclusion.

Importantly, in the event of a spillover leading to sustained human-to-human transmission of HeV or NiV, proof of concept studies already exist for the sG subunit vaccine in two animal models of which one is a non-human primate. This is relevant to the US FDA animal rule that states where a medical countermeasure cannot be evaluated in humans, *in vivo* evaluation of a vaccine or therapeutic may be translated from the outcomes of work in two animal models. Finally, the experimental vaccine given to ferrets uses a formulation that is in clinical trials in humans.

Recrudescence in the form of encephalitis has been documented for both HeV and NiV and is thought to be due to persistence of the virus in some form within the central nervous system. A farmer from Mackay, Australia developed encephalitis 13 months after apparent recovery from acute meningencephalitis caused by HeV and died with evidence of HeV in the brain as detected by PCR, electron microscopy and immunohistochemistry [29]. In the initial NiV outbreak in Malaysia 7.5% of survivors went on to develop relapsing encephalitis and 3.4% suffered late-onset encephalitis months to years after recovery from the initial infection [30,31]. Recent studies have shown that HeV can infect the mouse brain via an anterograde route of infection, probably along the olfactory nerve [32] as also suggested for NiV in pigs [33]. This suggests that an effective henipavirus vaccine will need to suppress the initial phase of replication in the upper respiratory tract (summarized in [8]) to a level that prevents infection of olfactory sensory neurons as well as preventing the onset of viremia.

The HeVsG subunit vaccine has proved highly effective in suppression of virus replication and disease prevention, with duration of protection that is sufficient to make the formulation attractive to industry. A vaccine incorporating the HeVsG antigen has now been released for use in horses and its application and observed effectiveness, along with ongoing work in animal models including nonhuman primates, will enable a more rapid response to any future henipavirus spillover events that threaten to cause large scale outbreaks in humans.

## Methods

### Animals, accommodation, handling and biosafety

Eight male ferrets aged 12 – 18 months were used in each of 2 experiments. Those exposed to NiV at 20 days post vaccination (study 1) were housed in pairs in cages in a single room at PC-3 or 4 for the duration of the study. Those exposed to NiV at 413 days post vaccination (study 2) were housed in pairs in cages at PC2 until the time of virus exposure, when they were moved to a single room at BSL-4. Animal husbandry was carried out as described in [22], and animal husbandry methods and experimental design were endorsed by the Australian Animal Health Laboratory’s Animal Ethics Committee.

### Animal infection

All ferrets were exposed to 5000 TCID_50_ of a low passage isolate of NiV-Bangladesh/human/2004/Rajbari, R1 [34,35]. This represents 10 times the minimum infectious dose determined for this virus in ferrets. General clinical observations were documented daily before and after infection (pi). Animals were weighed while under sedation at the time of vaccinations, virus exposure, and at days 6, 8, 10 and 21 pi. Rectal temperature was also recorded at sedation to augment data derived remotely from the implanted temperature transponders. Ferrets were euthanized when reaching a previously determined endpoint or at 21 days pi. The humane endpoint was defined as rapidly progressive clinical illness of up to 2 days duration including fever and depression, possibly accompanied by increased respiratory rate or posterior paresis or ataxia. In susceptible animals, this typically occurs within the first 10 days after viral challenge. In preliminary studies, these signs were found to correlate with the requirement to euthanize ferrets on subsequent days on humane grounds; thus, they have been utilized as surrogates for lethality.

### Vaccine immunogen preparation

A human codon optimized HeV soluble glycoprotein G (sG) construct was used to produce recombinant HeVsG as described [22]. CpG oligodeoxynucleotide (ODN) 2007 (TCGTCGTTGTCGTTTTGTCGTT) containing a fully phosphorothioate backbone was purchased from Invivogen (San Diego, CA, USA) and Alhydrogel^TM^ was purchased from Accurate Chemical & Scientific Corporation (Westbury, NY, USA). Vaccine doses containing fixed amount of CpG ODN 2007 and varying amounts of HeVsG and aluminum ion (at a weight ratio of 1:25) were formulated as follows: 4 μg sGHeV, 100 μg aluminium ion and 150 μg of CpG ODN 2007; 20 μg dose: 20 μg sGHeV, 500 μg aluminium ion and 150 μg of CpG ODN2007; and 100 μg dose: 100 μg HeVsG, 2.5 mg aluminium ion and 150 μg of CpG ODN 2007. For all doses, Alhydrogel^TM^ and sGHeV were mixed first before CpG ODN 2007 was added. Each vaccine dose was adjusted to 1 ml with PBS and mixtures were incubated on a rotating wheel at room temperature for at least 2–3 h prior to injection.

### Immunisation

For both studies, ferrets were divided randomly into 4 groups of 2, with each group receiving vaccine or adjuvant alone as follows: study 1 – 1–0 and 2–0 received adjuvant only, 3–4 and 4–4 were vaccinated with 4 μg HeVsG, 5–20 and 6–20 with 20 μg HeVsG and 7–100 and 8–100 with 100 μg HeVsG. Similarly for study 2, ferrets numbered 9–0 and 10–0, 11–4 and 12–4, 13–20 and 14–20, 15–100 and 16–100 received 0, 4, 20 and 100 μg HeVsG respectively. The vaccine was administered subcutaneously with a priming dose at day 0 and a booster dose 20 days later.

### Sample collection

Nasal washes, oral and rectal swabs and blood samples both in EDTA and for serum preparation, were taken at the time of vaccination and virus exposure, then at days 6, 8, 10 and 21 pi as described in [22]. Urine was collected only at post mortem examination when tissues were also collected for virus isolation, viral genome detection, histology, and immunohistology as described in [22]. These tissues included adrenal gland, bladder, brain, olfactory pole, heart, kidney, liver, apical lung lobe, diaphragmatic lung lobe, bronchial lymph node, retropharyngeal lymph node, spleen and testes.

### Sample analysis

TaqMan PCR assay for the detection of the NiV N gene, immunohistochemical evaluation using a rabbit polyclonal antibody raised against the NiV N protein [36], histology, serum neutralization titres against NiV and virus isolation were carried out according to [22].

## Competing interests

CCB is a United States federal employee and an inventor on pending United States patents and Australian patent 2005327194, pertaining to soluble forms of Hendra and Nipah G glycoproteins; assignees are The United States of America as represented by the Department of Health and Human Services (Washington, DC), Henry M. Jackson Foundation for the Advancement of Military Medicine, Inc. (Bethesda, MD).

## Authors’ contributions

JAP designed the study, processed animal tissues, carried out virus isolation and serology and drafted the manuscript. RK processed animal tissues, carried out real-time PCR, virus isolation and serology. RA and JH carried out the animal infection studies. FL read histopathology and immunohistopathology slides. JRW extracted viral RNA from animal tissues and fluids. JP processed tissues for histopathology and immunohistopathology. LFW conceived the study and edited the manuscript. CCB conceived the study, provided funds and edited the manuscript. DM conceived and designed the study, supervised animal infection studies, provided veterinary pathology expertise and edited the manuscript. All authors read and approved the final manuscript.
